# Spatial Trends in *Salmonella* Infection in Pigs in Spain

**DOI:** 10.3389/fvets.2020.00345

**Published:** 2020-06-23

**Authors:** Kendy Tzu-yun Teng, Marta Martinez Avilés, Maria Ugarte-Ruiz, Carmen Barcena, Ana de la Torre, Gema Lopez, Miguel A. Moreno, Lucas Dominguez, Julio Alvarez

**Affiliations:** ^1^VISAVET Health Surveillance Center, Universidad Complutense, Madrid, Spain; ^2^Center for Animal Health Research, National Institute of Agricultural and Food Research and Technology, Madrid, Spain; ^3^Ministerio de Agricultura, Alimentación y Medio Ambiente (Spain), Madrid, Spain; ^4^Department of Animal Health, Faculty of Veterinary Medicine, Universidad Complutense, Madrid, Spain

**Keywords:** *Salmonella*, pigs, spatial analysis, spatial modeling, BYM model, BYM2 model, Stan, Bayesian penalized regression

## Abstract

*Salmonella* is one of the most important foodborne pathogens worldwide. Its main reservoirs are poultry and pigs, in which infection is endemic in many countries. Spain has one of the largest pig populations in the world. Even though *Salmonella* infection is commonly detected in pig farms, its spatial distribution at the national level is poorly understood. Here we aimed to report the spatial distribution of *Salmonella*-positive pig farms in Spain and investigate the presence of potential spatial trends over a 17-year period. For this, data on samples from pigs tested for *Salmonella* in 2002–2013, 2015, 2017, and 2019 as part of the Spanish Veterinary Antimicrobial Resistance Surveillance program, representing 3,730 farms were analyzed. The spatial distribution and clustering of *Salmonella*-positive pig farms at the province level were explored using spatial empirical Bayesian smoothing and global Moran's *I*, local Moran's *I*, and the Poisson model of the spatial scan statistics. Bayesian spatial regression using a reparameterized Besag-York-Mollié Poisson model (BYM2 model) was then performed to quantify the presence of spatially structured and unstructured effects while accounting for the effect of potential risk factors for *Salmonella* infection at the province level. The overall proportion of *Salmonella*-positive farms was 37.8% (95% confidence interval: 36.2–39.4). Clusters of positive farms were detected in the East and Northeast of Spain. The Bayesian spatial regression revealed a West-to-East increase in the risk of *Salmonella* infection at the province level, with 65.2% (50% highest density interval: 70–100.0%) of this spatial pattern being explained by the spatially structured component. Our results demonstrate the existence of a spatial variation in the risk of *Salmonella* infection in pig farms at the province level in Spain. This information can help to optimize risk-based *Salmonella* surveillance programs in Spain, although further research to identify farm-level factors explaining this pattern are needed.

## Introduction

*Salmonella* infection is one of the most important foodborne zoonoses worldwide. There were 91,662 confirmed human salmonellosis cases in 2017 in the European Union (EU) (1) and ~1.2 million estimated cases occur every year in the US (2). Salmonellosis, characterized by acute onset of fever, abdominal pain, diarrhea, and nausea, is usually self-limiting. However, sometimes it can be life-threatening, especially in children, elderly and immunosuppressed patients, thus requiring antimicrobial therapy (3). This can be further complicated by the presence of antimicrobial-resistant strains (4).

Poultry is considered a major source of foodborne salmonellosis globally (5), but pork and pork products are also implicated in a large number of cases in many countries, including Spain (6, 7). Two of the most common *Salmonella* serotypes in pigs, *S*. Typhimurium and its monophasic variant (1,4,[5],12:i:-), were among the top serotypes associated with human salmonellosis in Spain (1). Moreover, the European Food Safety Authority (EFSA) baseline reports on *Salmonella* infection in fattening and breeder pig farms in Europe demonstrated that Spain had one of the highest levels of infection in pigs among EU countries, further highlighting their potential role in the occurrence of human salmonellosis in Spain (8, 9).

Still, due to the absence of a national control/monitoring program for *Salmonella* in pigs in Spain, little is known about the spatial distribution of *Salmonella* infection in pigs in Spain. The only available data came from specific studies with a generally limited geographical and temporal scope (10–12). The Spanish Veterinary Antimicrobial Resistance Surveillance Network program (13), starting in 1997, has performed nationwide surveillance of antimicrobial resistance originating from foodborne bacteria, such as *Escherichia coli, Campylobacter* spp., and *Salmonella* spp. Active surveillance of antimicrobial resistance in *Salmonella* in healthy pigs has been conducted through this program since 2002. Even though pigs from over 164 farms were sampled every year at the abattoirs since the beginning of the program, the spatial distribution of positive farms has never been evaluated.

Geographic information systems and some spatial statistical analyses have been applied to epidemiological research on *Salmonella* in farm animals. These approaches have allowed the detection of patterns and clusters of infection and prediction of occurrence and risk of infection of *Salmonella* under different situations. *K*-function analysis (14, 15) and Moran's *I* (16, 17) have been used to detect spatial clustering in *Salmonella* infection in farm animals, and a Gaussian kernel function has been used to predict the occurrence of *Salmonella*-infected dairy cattle and pig herds (18, 19). Using human cases, Simpson et al. (21) used the Besag-York-Mollié (BYM) hierarchical model (20) to map the cases of *S*. Wangata and *S*. Typhimurium in New South Wales, Australia (21). However, the usefulness of this method to better understand *Salmonella* infection in pigs seems not to be explored yet.

A BYM model contains two spatial random effects, often called spatially structured and unstructured components. The structured component has an intrinsic conditional autoregressive (CAR) prior that takes the geographical contiguity into account (correlated heterogeneity). The geographical contiguity is described by the neighborhood relationships between each pair of areas and a full spatial dependency. The unstructured one is a random effect for non-spatial heterogeneity at the same area level as the structured component. Riebler et al. (22) proposed a parameterized BYM model—the BYM2 model to address some limitations of the original BYM model. Briefly, the BYM2 model adopts the penalized complexity framework that favors a model whose parameters have clear interpretations and thus facilitates the use of sensible hyperparameters in the model (23). The BYM2 model combines the two spatial components in the original BYM model into a single spatial component and allows a parameter to describe the proportion of the variance explained by the structured component.

In the current study, we aimed to evaluate the spatial distribution and potential spatial trends of *Salmonella* infection in pig farms in Spain. To do so, we analyzed the data on *Salmonella* detection in samples from pigs across a 17-year period, derived from the Spanish Veterinary Antimicrobial Resistance Surveillance Network program, using several spatial analytical techniques, including a BYM2 model.

## Materials and Methods

### Study Population and Data Collection

Data on samples collected for monitoring antimicrobial resistance in *Salmonella* in pigs from 2002 to 2013, 2015, 2017, and 2019 (sampling was conducted every two years since 2015) were derived from the database of the Spanish Veterinary Antimicrobial Resistance Surveillance Network program. In the program, samples from fattening pigs were randomly collected in abattoirs selected based on their slaughter capacity. Each year, selected abattoirs altogether added up to more than 50% of the national slaughter capacity and were located in no less than half of the autonomous communities of the country. The total number of pigs sampled from each abattoir was proportional to their slaughter capacity and was randomly allocated to farm batches being culled on the sampling date. Animal samples consisted of at least 25 g of the content of caecum from two pigs selected at random from those coming from the same farm, except for 2011 when at least 15 g of ileo-caecal lymph nodes of one animal per farm were collected. Samples were collected by trained personnel, put in a clean container, and stored at refrigeration (3–8°C) until being sent to the laboratory within the next 36 h. *Salmonella* culture was performed immediately after reception.

### Bacteriology

*Salmonella* isolation was performed according to ISO 6579:2002/Amd 1:2007, the method recommended by the European Union Reference Laboratory for *Salmonella* in fecal and environmental samples [15]. Briefly, samples were cultured in buffered peptone water (BPW, 1/10 dilution; bioMérieux, Marcy-l'Étoile, France), followed by incubation at 37 ± 1°C for 18 ± 2 h. Modified semi-solid Rappaport-Vassiliadis (MSRV; Becton Dickinson France, Le Pont-de-Claix, France) agar plates were then inoculated with three drops (i.e., 0.1 ml) of BPW culture. Plates were incubated at 41.5 ± 1°C for 24 ± 3 h and, if negative, incubated for an additional 24 ± 3 h. Suspected growth of *Salmonella* was confirmed by plating out to both Xylose Lysine Desoxycholate agar (XLD; bioMérieux) and on chrom ID™ *Salmonella* agar (SM ID2; bioMérieux) for incubation during 24 ± 3 h at 37 ± 1°C.

Columbia 5% sheep blood agar (bioMérieux) was used for the incubation of colonies of presumptive *Salmonella* that were subcultured for 24 ± 3 h at 37 ± 1°C. All *Salmonella* isolates were confirmed by a commercial, biochemical method Enterotube™ II (BD BBL™; Becton Dickinson GmbH, Heidelberg, Germany). Serological typing was performed based on the White-Kauffmann-Le Minor scheme (24).

### Data on Potential Risk Factors (Covariates)

The potential of production-related characteristics to explain at least part of the observed spatial patterns of the risk of *Salmonella* infection in pig farms was assessed in the Bayesian spatial modeling (22 variables, Table 1) at the province level. These included (a) the number, the proportion, and the density (average number per square kilometer) of pigs belonging to different production categories (i.e., piglets, weaners, fattening pigs, gilts, sows, and boars) in each province and (b) the number and density of pig farms in each province. The pig-related information from 2005 to 2019 was collected from the website of the Ministry of Agriculture, Fisheries, and Food of Spain, and averaged over the study period for each province. The information about pig farm distribution was only available for 2016.

**Table 1:** Univariable generalized linear regression results for the risk of *Salmonella* infection in pigs at the province level in Spain from 2002 to 2013, 2015, 2017, and 2019.

**Name**	**Mean**	**Standard deviation**	**95% posterior probability**	**Change in risk with every specified unit of increase (95% credible interval)**
Number of farms	0.08	0.03	(0.03 to 0.13)	1.010 (1.004 to 1.016)^1^
Density of farms (per km^2^)	0.07	0.02	(0.03 to 0.12)	1.014 (1.005 to 1.023)^2^
Number of fattening pigs	0.06	0.02	(0.03 to 0.10)	1.002 (1.001 to 1.003)^3^
Number of sows	0.06	0.02	(0.01 to 0.10)	1.010 (1.002 to 1.018)^3^
Number of piglets	0.05	0.02	(0.01 to 0.08)	1.002 (1.000 to 1.004)^3^
Number of weaners	0.06	0.02	(0.03 to 0.09)	1.003 (1.001 to 1.005)^3^
Number of gilts	0.05	0.02	(0.01 to 0.09)	1.040 (1.009 to 1.068)^3^
Number of boars	0.04	0.04	(−0.04 to 0.11)	1.262 (0.787 to 1.953)^3^
Total number of pigs	0.06	0.02	(0.02 to 0.10)	1.001 (1.000 to 1.001)^3^
Proportion of fattening pigs	0.08	0.04	(0.00 to 0.15)	1.009 (1.001 to 1.018)^2^
Proportion of sows	−0.11	0.06	(−0.24 to 0.00)	0.989 (0.976 to 1.000)^2^
Proportion of piglets	−0.08	0.03	(−0.15 to −0.02)	0.989 (0.981 to 0.997)^2^
Proportion of weaners	0.09	0.03	(0.02 to 0.15)	1.016 (1.004 to 1.028)^2^
Proportion of gilts	−0.06	0.06	(−0.19 to 0.04)	0.976 (0.929 to 1.017)^2^
Proportion of boars	−0.09	0.06	(−0.22 to 0.03)	0.799 (0.566 to 1.091)^2^
Density of fattening pigs	0.07	0.02	(0.04 to 0.11)	1.003 (1.002 to 1.005)^4^
Density of sows	0.05	0.02	(0.01 to 0.09)	1.011 (1.002 to 1.020)^4^
Density of piglets	0.05	0.02	(0.01 to 0.08)	1.002 (1.000 to 1.004)^4^
Density of weaners	0.08	0.02	(0.04 to 0.12)	1.005 (1.002 to 1.008)^4^
Density of gilts	0.06	0.02	(0.01 to 0.10)	1.050 (1.012 to 1.089)^4^
Density of boars	0.05	0.03	(−0.02 to 0.11)	1.643 (0.813 to 3.211)^4^
Density of pigs	0.07	0.02	(0.03 to 0.11)	1.001 (1.000 to 1.002)^4^

1 *Every 100 farms of change*.

2 *Every 1% of change*.

3 *Every 10,000 animals of change*.

4 *Every 1 unit of change*.

### Statistical Analyses

Data cleaning, manipulation, and analyses were performed in Microsoft Excel 2013 (Microsoft Corp.), and R program version 3.5.2 (25) in RStudio interface version 1.2.1330 (26). Descriptive analyses were facilitated by “tidyverse” package (27). The location of all farms from which the sampled pigs originated was available at the province level and used for the following analyses.

The overall, yearly and province-level proportion of *Salmonella*-positive farms was calculated. Empirical Bayesian smoothing was then performed on the proportion of *Salmonella*-positive farms at the province level to incorporate information on the sample size and the proportion of *Salmonella*-positive farms of neighboring provinces by using the “spdep” package (28). Gabriel Graph was used to describe the neighboring relationships between the provinces throughout the study. In addition, the overall and yearly proportion of farms positive to specific *Salmonella* serotypes over the total number of positive farms were calculated for serotypes with >50 isolates over the study period. Empirical Bayesian smoothing, as previously described, was also used to map the proportion of farms positive to these *Salmonella* serotypes over the total number of positive farms at the province level.

The presence of global and local spatial autocorrelation in the spatial distribution of *Salmonella* positive farms was explored using global and local Moran's statistics (29, 30). A global and local Moran's *I*-tests were run on the standardized residuals of a Poisson model using the number of positive farms in each province as the outcome and the expected number of positive farms as the offset with the “spdep” package (28). The significance of the global Moran's *I* statistic was estimated through 999 Monte Carlo simulations in which the residuals were randomly shuffled across provinces. For local Moran's *I*, the *P*-values were calculated using the expectation and variance and corrected with the method described in Benjamini and Hochberg (31). The significance level for all the tests in the current study was set at 0.05.

Additionally, the Poisson model of the scan statistic was also applied to detect the presence of provinces with an increased risk of *Salmonella* infection using the centroid of each province as the point location, facilitated by the “SpatialEpi” package (32, 33). The scan statistic detects the maximum likelihood ratio between the value inside and outside a searching window over the likelihood function under the null hypothesis of complete spatial randomness (32). The search was performed using circular spatial moving windows that contained up to 25% or 50% of the total population alternatively. The pseudo *P*-values of the most likely clusters were generated by comparing the observed risk in the windows with the expected, generated through 999 Monte Carlo simulations in which the risks at each location were randomly allocated.

Bayesian spatial modeling to assess the associations between the risk of *Salmonella* infection at the province level and the available covariates was performed in Rstudio using Stan and associated packages (34). The number of observed *Salmonella*-positive pig farms in different provinces was assumed to follow a Poisson distribution, with the expected number of positive farms in each province as the offset. Regression models were fitted using the “brms” package (35). To explore the directionality of the association between available covariates and the outcome, univariable non-spatial models were first fitted introducing alternatively each of the covariates. For covariate selection, the predictive projection technique proposed by Piironen et al. was implemented using the “projpred” package (36, 37). The selection process contained two steps. First, a Bayesian penalized regression model with a regularized horseshoe prior and all the covariates was constructed as the reference model that warranted a good prediction ability. Penalized regression is a statistical technique designed to avoid overfitting, especially in cases of a large number of covariates (38). This is achieved through the introduction of a penalty term that shrinks small coefficients toward zero while leaving large coefficients large. The implement of penalized regression is rather intuitive within a Bayesian framework as a penalty term can be included as a hyperprior, also called shrinkage prior (38). Many shrinkage priors have been proposed, and a regularized horseshoe prior was chosen for the current study due to the advantages discussed in Piironen and Vehtari (39). Second, the covariates in the best model for each submodel size were identified by decreasing the Kullback–Leibler divergence from the reference model to the projected submodel using a forward stepwise addition. A submodel with the minimal subset of these covariates which had similar predictive power as the reference model, judged by the mean log predictive density and the root mean square error, was selected.

The BYM2 Poisson model including the selected covariates and the spatial components (Equation 1) was then fitted (22). To examine the suitability of alternative distributions to fit the data, another three BYM2 models were fitted with different likelihoods (i.e., zero-inflated Poisson, negative binomial and zero-inflated negative binomial) followed by model selection using Bayesian leave-one-out cross-validation (40, 41). The default priors specified in the “brms” package were used in the current analyses. Sampling was drawn from four Markov chains with 1,000 iterations. The results final model reported in the next section were sampled from four Markov chains with 3,000 iterations. Half of the iterations were for warm-up (i.e., burn-in) and not used for inference.

Markov chain Monte Carlo diagnostics for the final model were performed with (a) the potential scale reduction statistic (R^) (42), (b) the ratio of the effective sample size to the total sample size drawn from the posterior distribution, and (c) trace plots of Markov chain Monte Carlo generated through the “bayesplot” package (43). Residual check and posterior predictive checks were also performed using the “bayesplot” package (43, 44). The highest density interval of the posterior distribution was estimated using the “bayestestR” package (45).

**Equation 1**. Spatial component in a BYM2 model.

$$\begin{array}{l} T=\frac{1}{\sqrt{T_{t}}}\left(\sqrt{p/f}*S+\sqrt{1-p}\right) \end{array}$$

- *T*:*total spatial component*,
- τ_*t*_:*precision for the total spatial component*; $\frac{1}{\sqrt{T_{t}}}$ is the overall standard deviation,
- *pϵ* [0, 1]: the proportion of the variance explained by the spatially structured component,
- *f*: scaling factor,
- *S*: spatially structured component,
- *U*: spatially unstructured component.

For the variables potentially associated with the *Salmonella* detection (see below), the existence of major changes in their spatial distribution over the study period was evaluated using Friedman tests on the province-level yearly data, followed by pairwise Wilcoxon rank sum tests with *P*-values adjusted for multiple comparisons (46).

## Results

Up to 3,730 samples collected over the 15 years in which sampling was conducted, representing the same number of farms, were included in the current study, with an average of 249 (range: 163–384) samples per year. The number of abattoirs where the samples were collected each year, except for 2019 when this information was not available, ranged between 7 and 20. A median of 18 samples (interquartile interval: 11–29, range: 1–60) were collected from each abattoir each year during the study period. Abattoirs were located in 11 out of the 18 autonomous communities in Spain, and 977 (29.2%), 670 (20.0%) and 455 (13.3%) samples came from abattoirs in Cataluña, Castilla La Mancha and Murcia, respectively (Supplementary File 1). The sampled farms were located in 43 out of the 52 provinces in Spain; 502 (13.5%) were from Murcia, 371 (9.95%) from Huesca, and 334 (9.0%) from Lleida.

**Figure 1:**
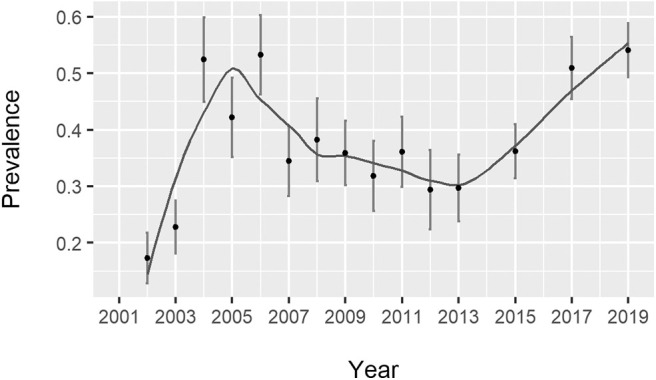
Annual proportion of *Salmonella*-positive farms in Spain from 2002 to 2013, 2015, 2017, and 2019. The line was smoothed by a locally estimated scatterplot smoothing with a span of 0.5.

**Figure 2:**
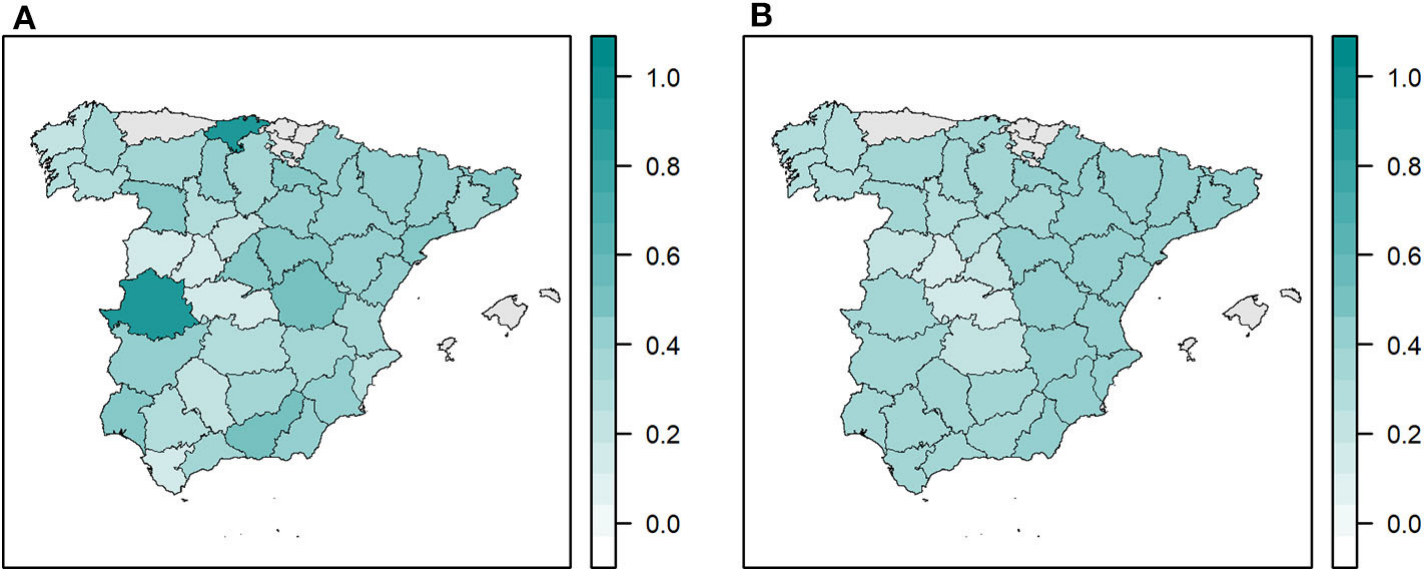
Proportion of *Salmonella*-positive farms at the province level in Spain from 2002 to 2013, 2015, 2017, and 2019. **(A)** Raw proportion. **(B)** Spatially adjusted proportion using empirical Bayesian smoothing.

A total of 1,409 of the 3,730 samples were positive, yielding an overall percentage of *Salmonella*-positive farms of 37.8% (95% confidence interval [CI]: 36.2–39.4). This percentage peaked between 2004 (54.2%) and 2006 (53.3%), declined to 29.7% in 2012, and increased again to 54.1% in 2019 (Figure 1). The raw and smoothed proportion of *Salmonella*-positive farms at the province level is shown in Figure 2. In the eleven provinces with more than 100 samples, the spatially adjusted proportion ranged from 17.8 % (95% CI: 13.1–22.2) in Toledo to 44.8% (95% CI: 35.4–54.2) in Almeria.

**Figure 3:**
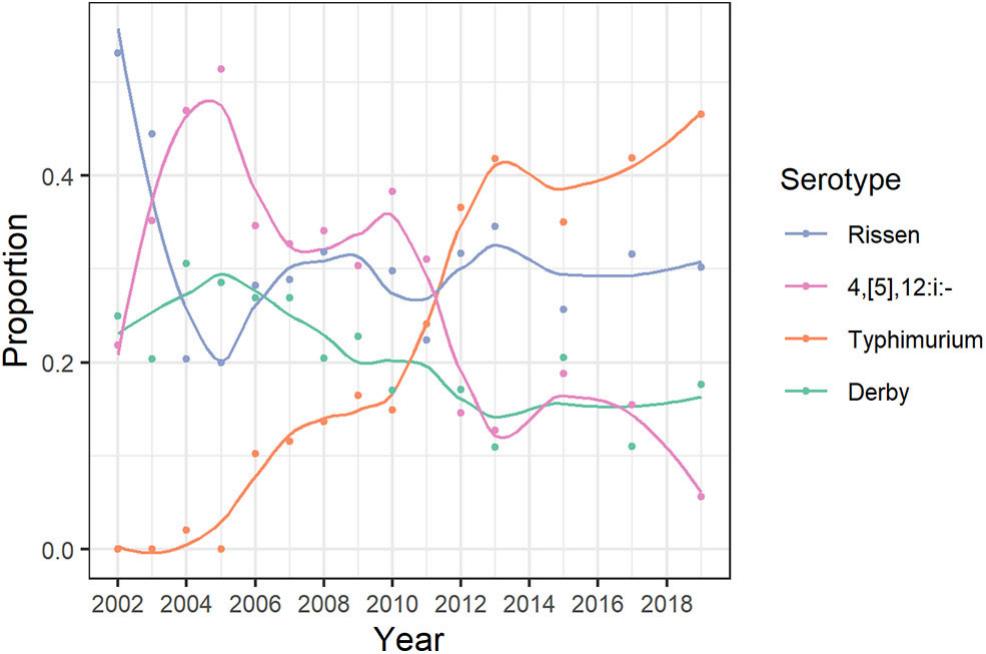
Changes in the proportion of *Salmonella* isolates recovered through the Spanish Veterinary Antimicrobial Resistance Surveillance Network program belonging to one of the four most common serotypes collected in pigs in Spain from 2003 to 2013, 2015, 2017, and 2019. The lines were smoothed by a locally estimated scatterplot smoothing with a span of 0.5.

**Figure 4:**
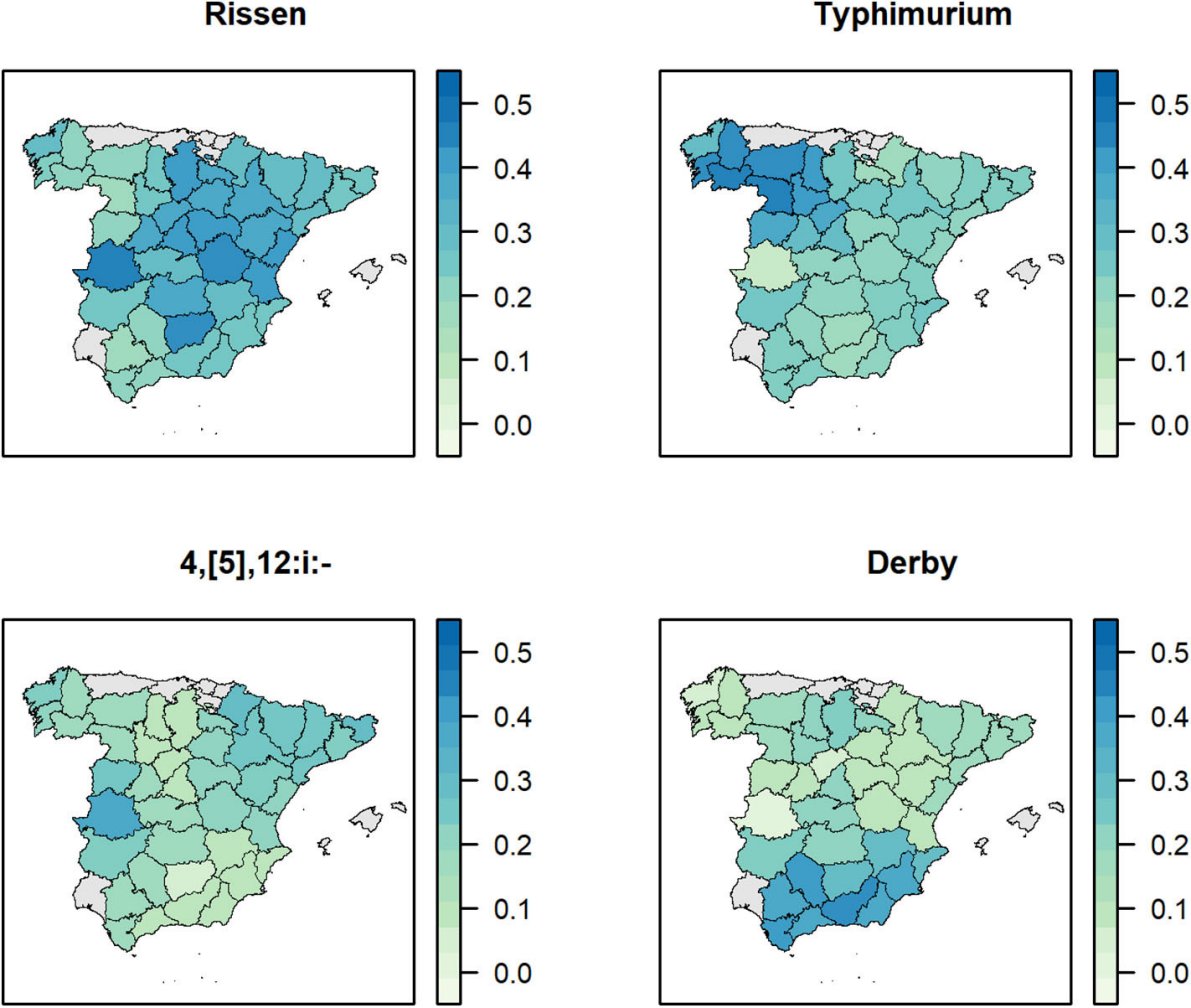
Empirical Bayesian smoothed proportions of farms positive to the four most represented *Salmonella* serotypes collected through the Spanish Veterinary Antimicrobial Resistance Surveillance Network program in pigs in Spain from 2002 to 2013, 2015, 2017, and 2019.

The serotype of 1,360 (96.5%) out of the total 1,409 *Salmonella* isolates recovered was determined, yielding 64 distinct serotypes (Supplementary Table 1). The most represented were *S*. Rissen (313, 22.2%), *S*. 1,4,[5],12:i:- (265, 18.8%), *S*. Typhimurium (251, 17.8%), and *S*. Derby (207, 14.7%). The evolution of the proportion of isolates belonging to specific serotypes over the study period varied (Figure 3). The proportion of *S*. Typhimurium decreased after 2010, while the proportion of the *S*. 1,4,[5],12:i:- has consistently increased over the years. The proportion of *S*. Rissen remained consistently around 0.3 after 2005, and, for *S*. Derby, a decreasing trend was observed (from slightly lower than 0.3 to <0.2 from 2012 onwards). After the empirical Bayesian smoothing, the proportions of *Salmonella* isolates identified as the *S*. 1,4,[5],12:i:- and *S*. Derby were higher in the provinces of Northeast and South of Spain, respectively (Figure 4). Both the proportions of *S*. Rissen and the *S*. 1,4,[5],12:i:- were rather homogeneous across provinces in Spain but slightly lower at the northwest corner and the South of Spain, respectively.

**Figure 5:**
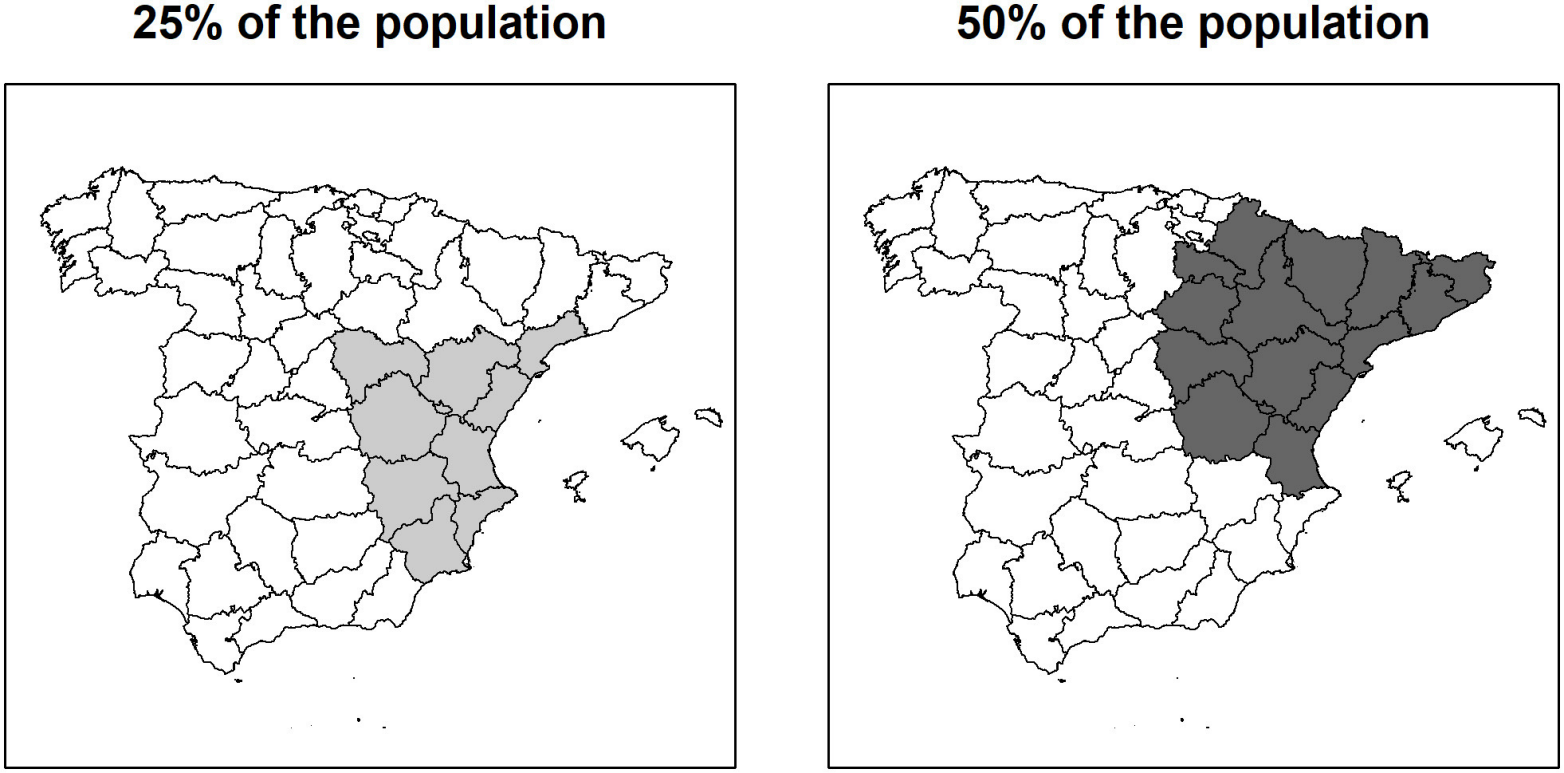
Provinces included in the significant, high-risk *Salmonella* clusters detected by the Poisson model of the spatial scan statistics using data collected through the Spanish Veterinary Antimicrobial Resistance Surveillance Network program in pigs in Spain from 2002 to 2013, 2015, 2017, and 2019.

No global (Moran's *I* = −0.02, *p* = 0.458) or local spatial autocorrelation was detected in the standardized residuals of the Poisson model. The spatial scan statistics identified local clusters with an increased risk of *Salmonella* infection in the East and Northeast of Spain (Figure 5). The observed-to-expected ratio between inside and outside of the significant clusters identified using a search window of maximum 25 and 50% population was 1.17 (*P* = 0.036) and 1.13 (*P* = 0.001), respectively.

According to the univariable models, provinces with a higher number or density of pig farms showed a higher risk of *Salmonella* infection (Table 1). In general, covariates related to the population of fattening pigs, weaners, sows or piglets were positively associated with the probability of finding positive *Salmonella* farms.

After predictive projection, only one covariate, the density of weaners, was selected to be included in the final Poisson BYM2 model (Table 2). More results of Bayesian penalized regression and predictive projection can be found in Supplementary File 2. In the Poisson model including only the density of weaners without the BYM2 component, the probability of finding positive *Salmonella* farms at the province level increased by 0.5% (95% credible interval [CrI]: 0.2–0.8%) with every increase in the weaner density. However, the effect of this covariate shrank to close to 0 after the inclusion of the BYM2 component (Table 2).

**Table 2:** Regression results from the final multivariable modeling for risk of *Salmonella* infection in pigs at the province level in Spain from 2002 to 2013, 2015, 2017, and 2019.

**Variable**	**Mean**	**Standard deviation**	**95% posterior probability**	**Change of risk with very 1 unit of increase (95% credible interval)**
Intercept	−0.07	0.05	−0.17 to 0.02	–
Density of weaners	−0.01	0.06	−0.13 to 0.10	−0.001 (−0.008 to 0.007)
Standard deviation of the spatial component	0.23	0.05	0.14 to 0.35	–
Proportion explained by the structured component	0.65	0.25	0.10 to 0.99	–

**Figure 6:**
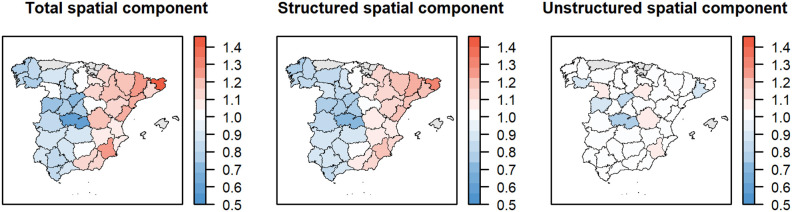
The total (left) spatial risk of *Salmonella* infection in pigs at the province level in Spain from 2002 to 2013, 2015, 2017, and 2019 according to the Poisson BYM2 model and the risk explained by the structured (center) and unstructured (right) spatial components.

The spatial component in the final BYM2 model suggested a West-East increasing risk of *Salmonella* infection at the province level in Spain. The exponentiated means of the spatial effects are shown in Figure 6. The mean of the standard deviation of the spatial component was 0.23 (Crl: 0.14–0.35), and, on average, 65.2% (median: 70.2%, 50% highest density interval: 70–100%) of the variance of the spatial component was explained by the structured component. No specific pattern in the unstructured spatial component was observed.

Markov chain Monte Carlo and model diagnoses are presented in Supplementary File 3.

The density of weaners at the province level did not experience major changes over the study period, with higher values reported consistently for provinces in the Northeast corner (Supplementary File 4). Still, the Friedman test revealed the existence of significant (*P* < 0.001) differences in the yearly values over time, although *post-hoc* tests revealed that differences were only due to values recorded in 2017 and 2019The test became non-significant (*P* = 0.071) when values from 2005 to 2015 were used.

## Discussion

Human salmonellosis outbreaks have been linked to pork and pork products in the past (1, 5, 7). In many regions and countries such as Northern and Western Europe and Japan, pork, and pork products are the second most common source for human salmonellosis after eggs and egg products (5). In Spain, pork and pork products have been shown to be one of the top-ranked sources for human salmonellosis (7). However, currently, there is no official control program of *Salmonella* in pig production in Spain (47). In the current study, we used several analytical techniques to explore the spatial distribution of *Salmonella* infection in pigs in Spain and generate information that can be used for surveillance of *Salmonella* in pigs in the future.

We detected an overall percentage of *Salmonella*-positive farms of 37.8% with great variation across the years. Several studies have reported even higher values of farm-level *Salmonella* prevalence in Catalonia (77.3% in 2000–2003), northeast Spain [94.1% in 2008–2009 (12)], and the entire country [43.1% in Spain in 2003–2004 (11)]. The lower values suggested by the current study could be partially due to the inclusion of feces from only two pigs per farm. Thus, the probability of detecting the presence of *Salmonella* at the farm level, particularly in farms with a low within-farm prevalence, was not as high as in the aforementioned studies. Nonetheless, the spatial heterogeneity found in the current study agrees with these previous results reporting higher prevalence values in the Northeast of the country, where a large proportion of Spanish pig population is located.

Our results indicated that the recent increase in the percentage of *Salmonella*-positive farms could be related to the increasingly reported *S*. 1,4,[5],12:i:-. During the study period, the proportion of isolates belonging to this serotype steadily rose and reached 0.47 in 2019, in parallel to the increase in the yearly percentage of *Salmonella*-positive farms (from 29.4 in 2012 to 54.1 in 2019). The importance of the *S*. 1,4,[5],12:i:- in public health highlights the need for continuous monitoring on its prevalence in pigs in Spain and the EU at large (48).

Several factors may affect the determination of the *Salmonella* status of a farm when samples are collected at the abattoir. Many studies have shown a noticeably higher prevalence of *Salmonella* in samples from slaughtered pigs than the prevalence of the samples from the farm (49, 50). This could be due to the stress generated by the process of harvest, transportation, and retention in the lairage, resulting in the recrudescence of latent carriers and/or an increase in the susceptibility of pigs to new infections (49). Moreover, the long feed withdrawal normally performed before transport might change the gut microbiota and increase the number of *Salmonella* in the fecal content (51). On the other hand, an increase in the diversity of serotypes detected in the abattoir compared to those recovered from the farms of origin has been also described. This could suggest exposure to additional contaminated environments such as trucks and lairages (49, 52). Therefore, the true prevalence of *Salmonella* infection at the farm level may be lower than the detected/apparent prevalence based on samples collected at abattoirs, and the diversity of serotypes could be the result of *Salmonella* from both pig farms and places involved in the process between harvest and slaughtering.

Our results showed a clear pattern suggesting a higher risk of *Salmonella* infection in the (North-)east than in the rest of Spain (Figure 6). The BYM2 model showed that, on average, 65.2% of the spatial effect could be explained by the underlying geographical location of the provinces, and the highest density interval included even higher values. We considered several pig population-related covariates in the analysis given that their heterogeneous spatial distribution was in agreement to some extent to the results of the spatial scan statistic (i.e., more pigs and higher densities in the Northeast of Spain). The univariable modeling results showed that many covariates, especially the number of farms and those related to fattening pigs and weaners, were indeed positively associated with the risk. However, only one covariate, the density of weaners, was retained after the variable selection process. This was expected as many covariates were correlated. Nonetheless, the effect of the density of weaners became close to 0 after the inclusion of the BYM2 component. This suggested that the observed spatial distribution of risk was better explained by the geographical contiguity of the provinces than by the density of weaners and other pig-related factors.

The software used here, Stan, is a highly-expressive probabilistic programming language that allows full Bayesian inference using Hamiltonian Monte Carlo samplers (34), which have been shown more efficient and robust than Gibbs and Metropolis samplers (53). Although many R packages to facilitate Bayesian modeling in Stan have been developed (35, 54), it is yet to be much explored in the field of veterinary epidemiology. With Stan and “brms” package (35), BYM2 model can be performed by regular R users (22).

To our knowledge, the BYM2 model has been little utilized in the field of veterinary science. The BYM2 model has advantages over the original and some of the reparameterized BYM models (22). Firstly, the two components in a BYM2 model can be seen independently from each other, resulting in better estimation for both of them (55). Secondly, BYM2 models facilitate parameters that have clear interpretations and thus the use of sensible hyperparameters (23). Additionally, as the scaling factor in BYM2 models is placed to take into account the underlying neighborhood structure, studies with different neighborhood structure now can use the same hyperprior in the models (22). Therefore, the future application of the BYM2 model in veterinary epidemiology may be encouraged. A BYM2 model can also be performed with INLA (22).

In the current study, we employed a relatively unexplored approach for variable selection in veterinary epidemiology—predictive projection with a Bayesian penalized regression model as the reference model. Shrinkage methods have been recommended when the ratio of the number of observations to the number of variables is ≤ 10 (56), and predictive projection is useful in determining the number of variables to be included in the model (36). This technique has several advantages. First, it requires less computational power than cross-validation and is less time-consuming than using either cross-validation or information criteria. Second, selection among many models using cross-validation may tend to overfit and thus result in choosing a suboptimal model (36).

Here, a number of different spatial analytic tools were applied, offering different results. While the global and local Moran's *I*-tests ran on the residuals of a Poisson model did not suggest the existence of a spatial pattern in the distribution of the *Salmonella* risk at the province level, the spatial scan statistic and the Bayesian spatial model showed the opposite. This finding suggests that, rather than making a conclusion of spatial independence based on one test, the application of more than one spatial analytic test, based on different hypotheses and assumptions, can provide a more complete picture.

The current study has some limitations. First, as the sample collection was conducted in abattoirs that have high slaughter capacity, the results might not be necessarily representative of the farms that did not (usually) send their pigs to those abattoirs. Second, the current study was conducted using secondary data. Therefore, it may face some common issues of using secondary data, such as out-of-date information, suboptimal sampling procedure for answering specific research questions, insufficient sample size, and lack of information that would be, otherwise, included. For example, the individual or within-farm prevalence of *Salmonella* infection could not be determined in the current study. Also, farm-specific information was not available so could not be included in the models. Farm-specific covariates such as farms types, and management and biosecurity information, will likely be associated with the outcome and allow prediction of the risk. Furthermore, for the multivariable modeling exercise, farm-related information was only available for 2016, and data of pig distribution was averaged across all years under study. Still, the exploration of the evolution of the pig distribution in Spain revealed no significant variation between provinces for at least most of the study period (2002–2015), suggesting that aggregating values across such a period would not result in a major loss of information. Lastly, it has been shown that the inclusion of a spatially-correlated component only after the covariate selection process may affect the results of the covariates in the model (57). This is observed in the current study. Ideally, the predictive projection should be conducted with the BYM2 component included. However, this is currently unavailable in the “projpred” package and thus was not done.

## Conclusion

The current study shows a notable increasing trend in the risks of *Salmonella* infection in pig farms located in provinces from West to East in Spain, evident still after the possible effect of the heterogeneous distribution of pigs in the country was accounted for. The increase in the percentage of *Salmonella* positive farms from 2012 and the *S*. 1,4,[5],12:i:- in Spain demonstrates the usefulness of surveillance to detect changes in the epidemiology of this foodborne pathogen in the animal reservoirs. We demonstrated the usefulness of Stan for various applications that are commonly pursued in a veterinary epidemiological study, such as covariate selection, model selection, and model fit assessment, as well as fitting a BYM2 model. The information generated by the current study can be used for risk-based *Salmonella* antimicrobial resistance surveillance programs in the future, so the probabilities of selecting positive farms and specific serotypes can be optimized. Although some temporal trends in the risk of *Salmonella* are shown in the current study, more data is needed to allow a better understanding of the spatial-temporal distribution and the evolution of *Salmonella* infection in pigs in Spain.

## Data Availability Statement

The datasets generated for this study are available on request to the corresponding author.

## Ethics Statement

The current work did not involve any live animal nor human data. No ethical approval was required as all isolates analyzed were retrieved through the ongoing Spanish national surveillance program on antimicrobial resistance, performed according to national and EU regulations.

## Author Contributions

KT and JA contributed conception and design of the study. KT, MM, MU-R, CB, AT, GL, MAM, LD, and JA participated in the generation, collection, and curation of the data. KT performed the statistical analysis. KT, MM, JA interpreted the results. JA provided supervision. MU-R, GL, and JA facilitated project administration. KT wrote the first draft of the manuscript. MU-R and JA wrote sections of the manuscript. All authors contributed to manuscript revision, read, and approved the submitted version.

## Conflict of Interest

The authors declare that the research was conducted in the absence of any commercial or financial relationships that could be construed as a potential conflict of interest.
