# Supplementary material for: Spatial Trends in Salmonella Infection in Pigs in Spain
Source: Front Vet Sci. 2020 Jun 23;7:345. doi: 10.3389/fvets.2020.00345 (PMC7325609; doi:10.3389/fvets.2020.00345)
Supplement: Supplementary file 1 [file Data_Sheet_1.docx]

**Supplementary File 1**. Methods for *Salmonella* isolation and serological typing.

*Salmonella* isolation was performed according to ISO 6579:2002/Amd 1:2007, the method recommended by the European Union Reference Laboratory for *Salmonella* in faecal and environmental samples [15]. Briefly, samples were cultured in buffered peptone water (BPW, 1/10 dilution; bioMérieux, Marcy-l’Étoile, France), followed by incubation at 37 ± 1°C for 18 ± 2 hours. Modified semi-solid Rappaport-Vassiliadis (MSRV; Becton Dickinson France, Le Pont-de-Claix, France) agar plates were then inoculated with three drops (i.e., 0.1 ml) of BPW culture. Plates were incubated at 41.5 ± 1°C for 24 ± 3 hours and, if negative, incubated for an additional 24 ± 3 hours. Suspected growth of *Salmonella* was confirmed by plating out to both Xylose Lysine Desoxycholate agar (XLD; bioMérieux) and on chrom ID^TM^ *Salmonella* agar (SM ID2; bioMérieux) for incubation during 24 ± 3 hours at 37 ± 1°C.

Columbia 5% sheep blood agar (bioMérieux) was used for the incubation of colonies of presumptive *Salmonella* that were subcultured for 24 ± 3 hours at 37 ± 1°C. All *Salmonella* isolates were confirmed by a commercial, biochemical method Enterotube ^TM^ II (BD BBL ^TM^; Becton Dickinson GmbH, Heidelberg, Germany). Serological typing was performed based on the White-Kauffmann-Le Minor scheme (1).

**Reference**

1. Grimont PA, Weill FX. Antigenic formulae of the Salmonella serovars. *WHO Collab Cent Ref Res Salmonella* (2007) **9**:1–166.
