# Supplementary material for: Spatial Trends in Salmonella Infection in Pigs in Spain
Source: Front Vet Sci. 2020 Jun 23;7:345. doi: 10.3389/fvets.2020.00345 (PMC7325609; doi:10.3389/fvets.2020.00345)

**Supplementary File 2.** Figures of the number of visited abattoirs and farms where the sampled pigs were from.

**Figure 1.** The number of abattoirs where the samples were collected (dotes) and the number of samples collected (colour) from each autonomous community through the Spanish Veterinary Antimicrobial Resistance Surveillance Network programme in pigs in Spain from 2002 to 2013, 2015 and 2017 (there was no information about the abattoirs in 2019).


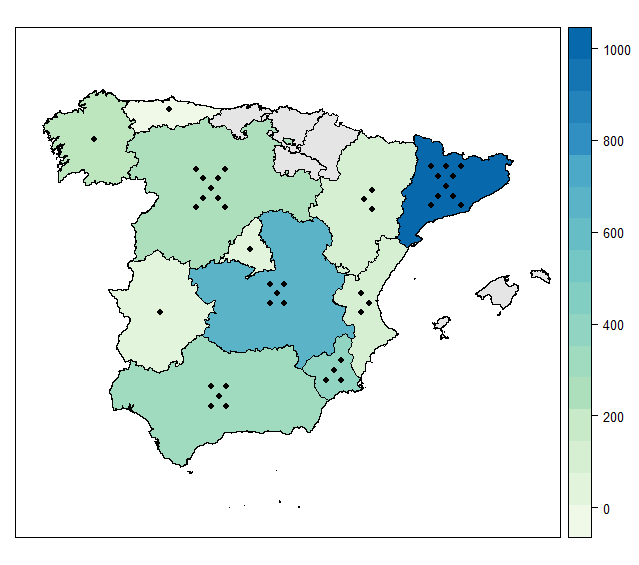


**Figure 2.** The number of farms where the sampled animals originated for each of the autonomous communities through the Spanish Veterinary Antimicrobial Resistance Surveillance Network programme in pigs in Spain from 2002 to 2013, 2015, 2017 and 2019. Autonomous communities in light grey denoted zero samples.


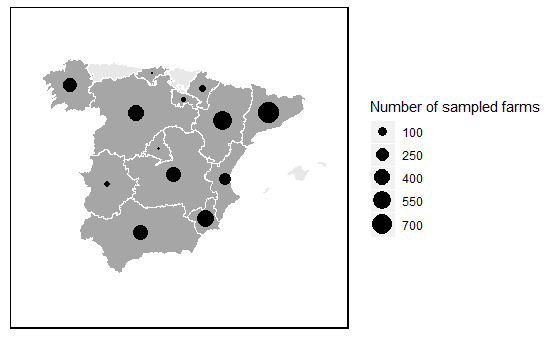

Supplement: Supplementary file 2 [file Data_Sheet_2.docx]
