# Supplementary material for: Spatial Trends in Salmonella Infection in Pigs in Spain
Source: Front Vet Sci. 2020 Jun 23;7:345. doi: 10.3389/fvets.2020.00345 (PMC7325609; doi:10.3389/fvets.2020.00345)
Supplement: Supplementary file 3 [file Data_Sheet_3.docx]

**Supplementary File 3. The results of Bayesian penalised regression and predictive projection**

**Table 1.** Bayesian penalised regression results.

| Name | Mean | Standard deviation | 95% posterior probability |
| --- | --- | --- | --- |
| Intercept | -0.07 | 0.04 | -0.15 to -0.002 |
| Number of farms | 0.00 | 0.02 | -0.026 to 0.057 |
| Density of farms (per km^2^) | 0.01 | 0.02 | -0.024 to 0.063 |
| Number of fattening pigs | 0.00 | 0.02 | -0.030 to 0.058 |
| Number of sows | 0.00 | 0.02 | -0.048 to 0.034 |
| Number of piglets | 0.00 | 0.02 | -0.039 to 0.038 |
| Number of weaners | 0.00 | 0.02 | -0.029 to 0.052 |
| Number of gilts | 0.00 | 0.02 | -0.031 to 0.047 |
| Number of boars | 0.00 | 0.02 | -0.042 to 0.033 |
| Total number of pigs | 0.00 | 0.02 | -0.037 to 0.051 |
| Proportion of fattening pigs | 0.01 | 0.03 | -0.017 to 0.082 |
| Proportion of sows | 0.00 | 0.02 | -0.060 to 0.025 |
| Proportion of piglets | -0.02 | 0.03 | -0.096 to 0.014 |
| Proportion of weaners | 0.01 | 0.02 | -0.016 to 0.074 |
| Proportion of gilts | 0.00 | 0.02 | -0.032 to 0.053 |
| Proportion of boars | 0.00 | 0.02 | -0.049 to 0.039 |
| Density of fattening pigs | 0.01 | 0.03 | -0.021 to 0.103 |
| Density of sows | 0.00 | 0.02 | -0.045 to 0.031 |
| Density of piglets | 0.00 | 0.02 | -0.054 to 0.031 |
| Density of weaners | 0.02 | 0.04 | -0.017 to 0.144 |
| Density of gilts | 0.00 | 0.02 | -0.040 to 0.041 |
| Density of boars | 0.00 | 0.02 | -0.039 to 0.034 |
| Density of pigs | 0.01 | 0.03 | -0.035 to 0.070 |

**Figure 1.** Plots of the changes in mean log predictive density (mlpd) and the root mean square error (rmse) relative to the reference model of the submodels.

**
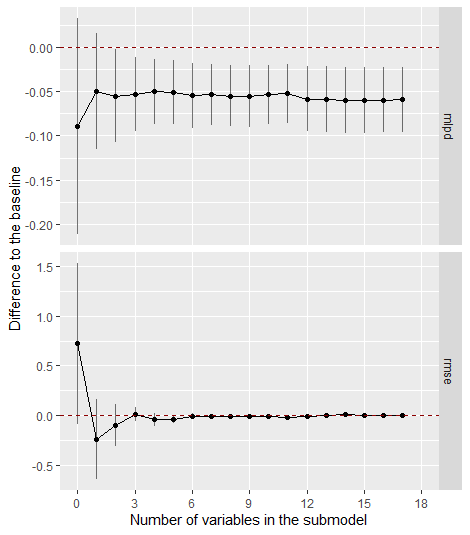
**

**Table 2.** Covariates in the order of forward stepwise addition in predictive projection (corresponding to Figure 1)

| Order | Covariate |
| --- | --- |
| 1 | Density of weaners |
| 2 | Proportion of piglets |
| 3 | Number of farms |
| 4 | Number of fattening pigs |
| 5 | Number of boars |
| 6 | Density of fattening pigs |
| 7 | Proportion of weaners |
| 8 | Proportion of fattening pigs |
| 9 | Total number of pigs |
| 10 | Density of pigs |
| 11 | Number of sows |
| 12 | Density of gilts |
| 13 | Proportion of sows |
| 14 | Density of farms |
| 15 | Proportion of gilts |
| 16 | Number of weaners |
| 17 | Density of boars |
