# Supplementary material for: Spatial Trends in Salmonella Infection in Pigs in Spain
Source: Front Vet Sci. 2020 Jun 23;7:345. doi: 10.3389/fvets.2020.00345 (PMC7325609; doi:10.3389/fvets.2020.00345)

**Supplementary File 4.** Markov chain Monte Carlo and model diagnoses.

**Figure 1.** Potential scale reduction statistic (Ȓ) of the parameters of the final Bayesian spatial model.


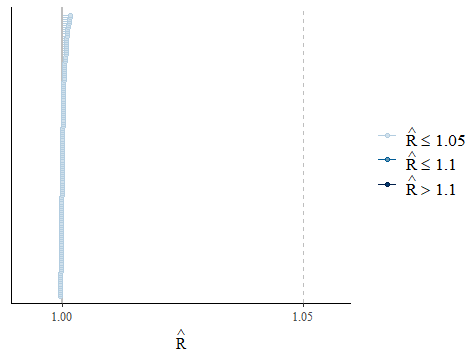


**Figure 2.** The ratio of the effective sample size to the total sample size drawn from the posterior distribution of the parameters of the final Bayesian spatial model.


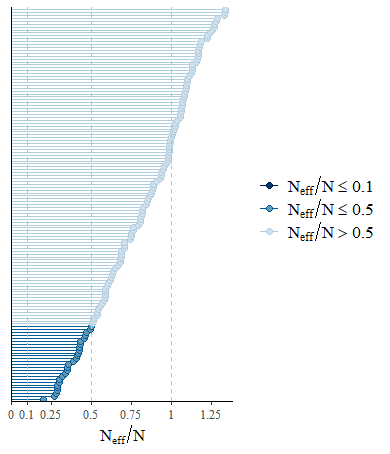


**Figure3.** Trace plots of Markov chain Monte Carlo of the parameters of the final Bayesian spatial model.


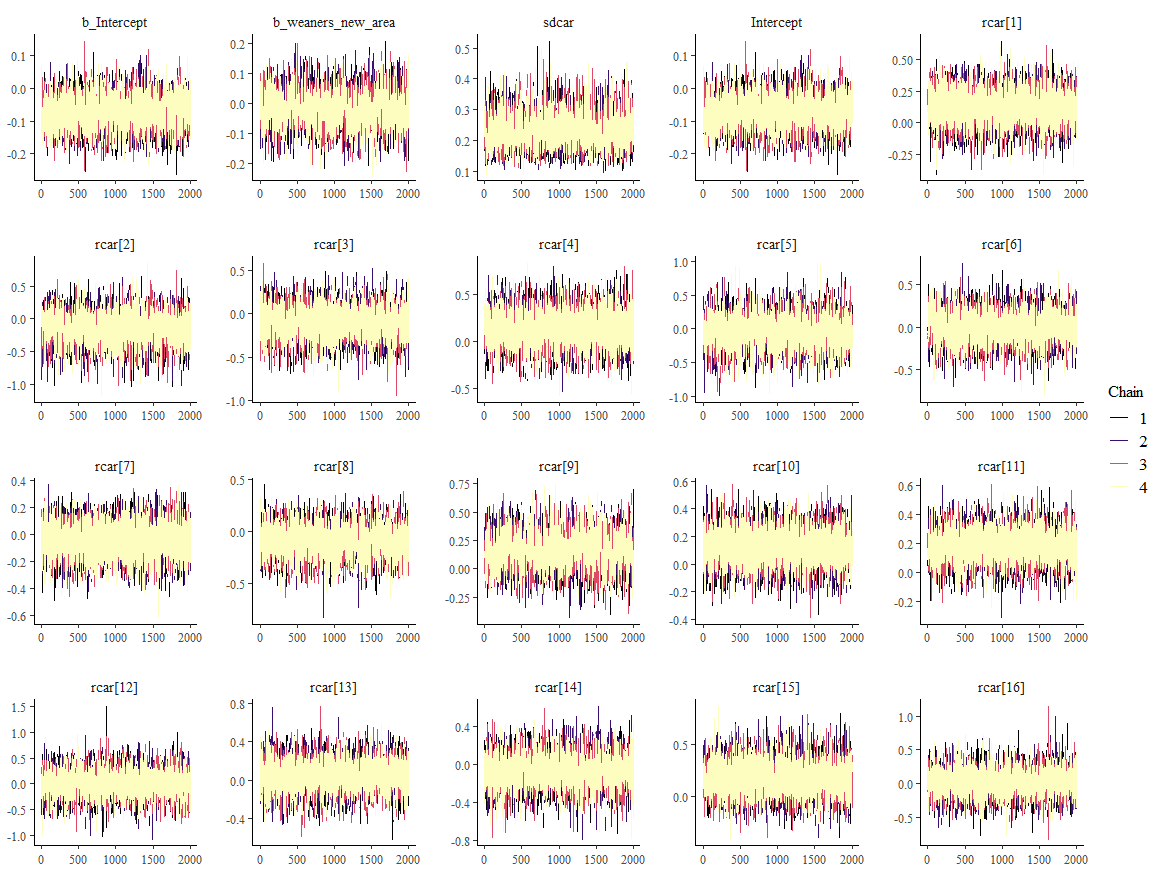


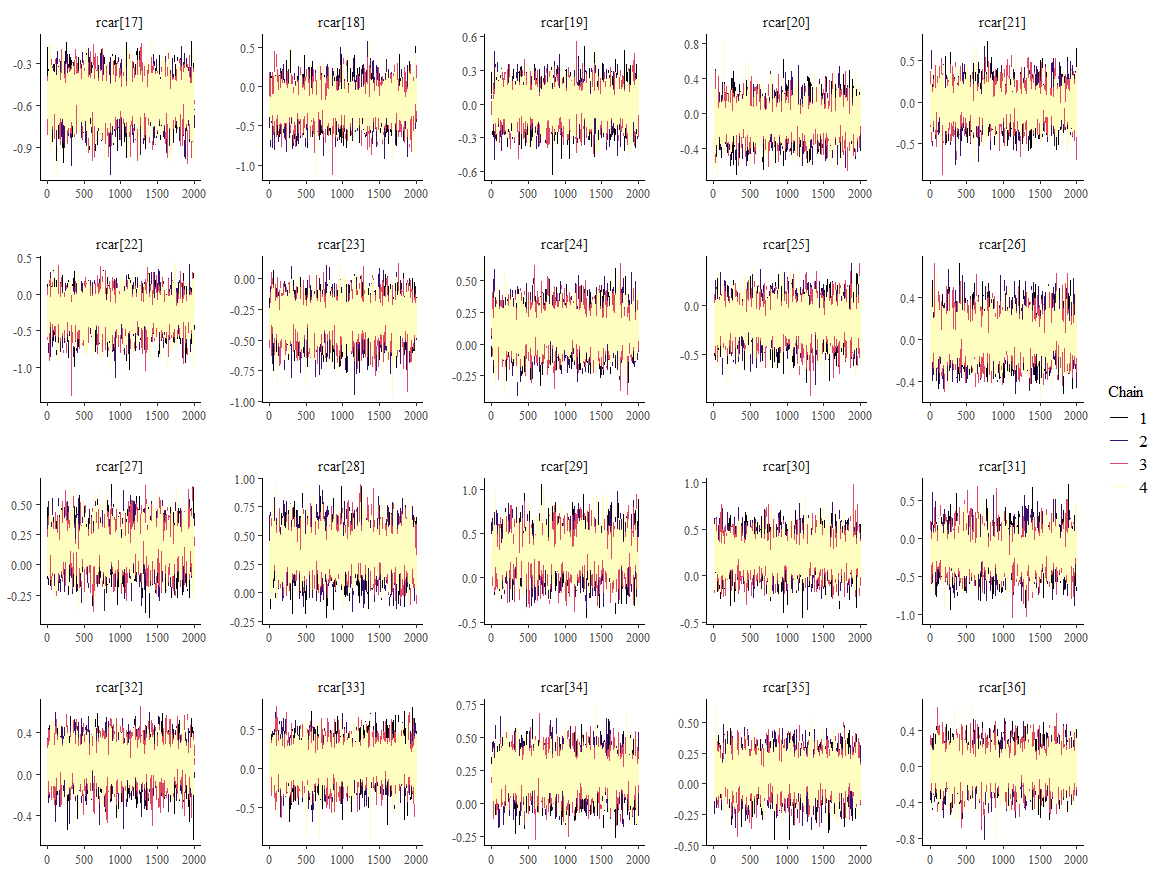


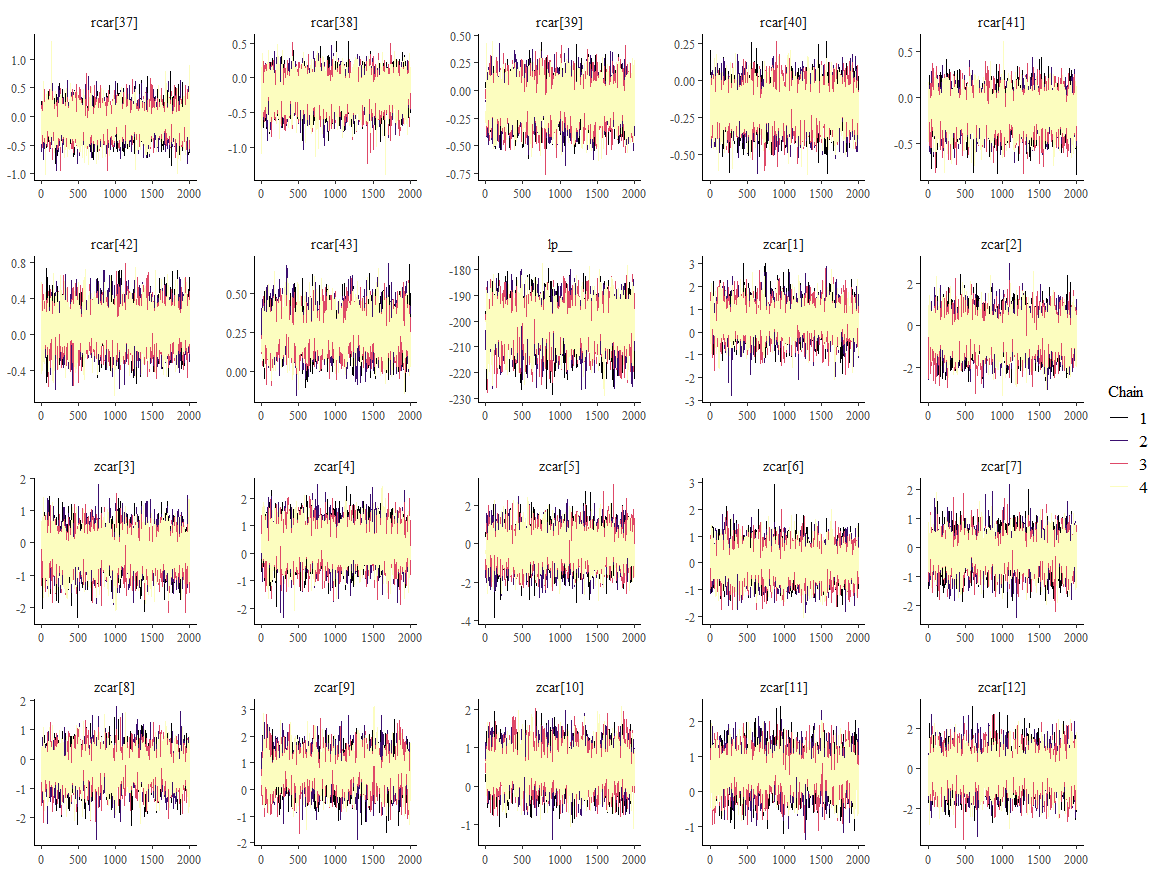


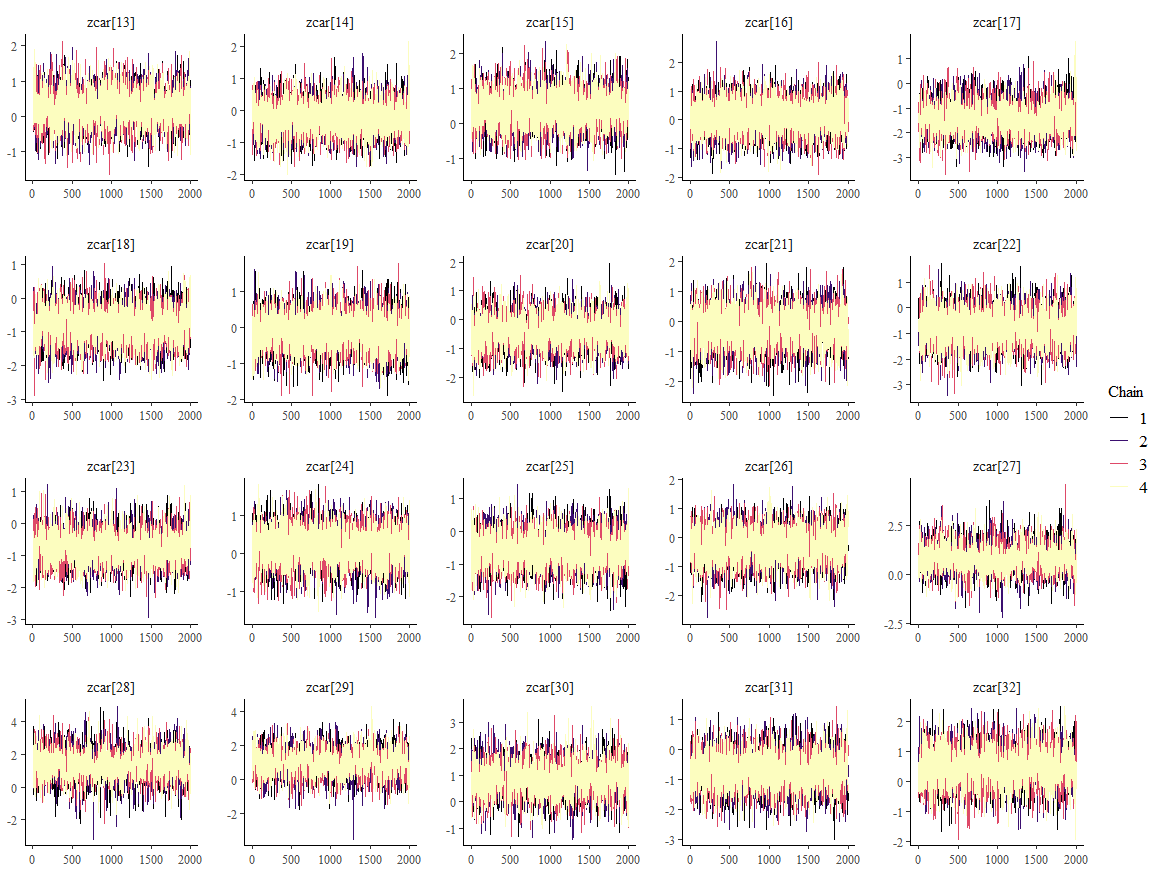


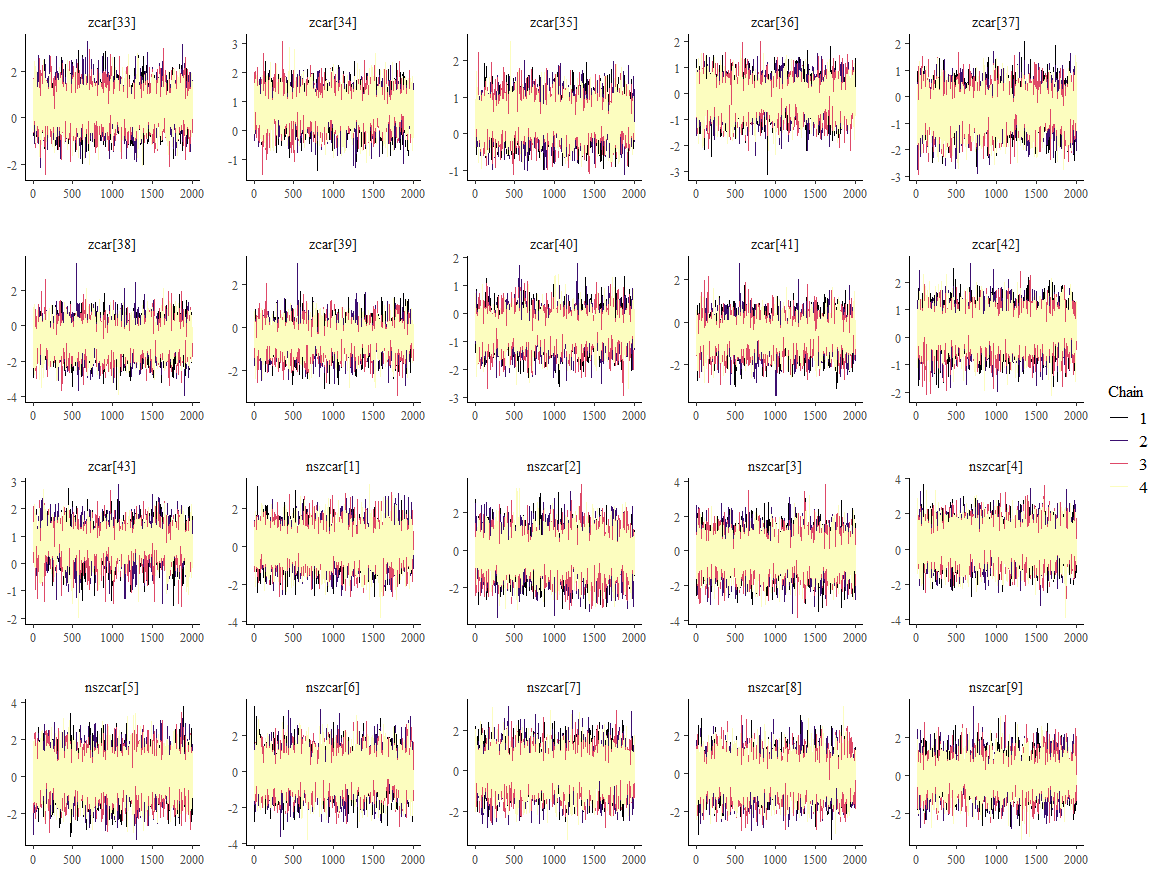


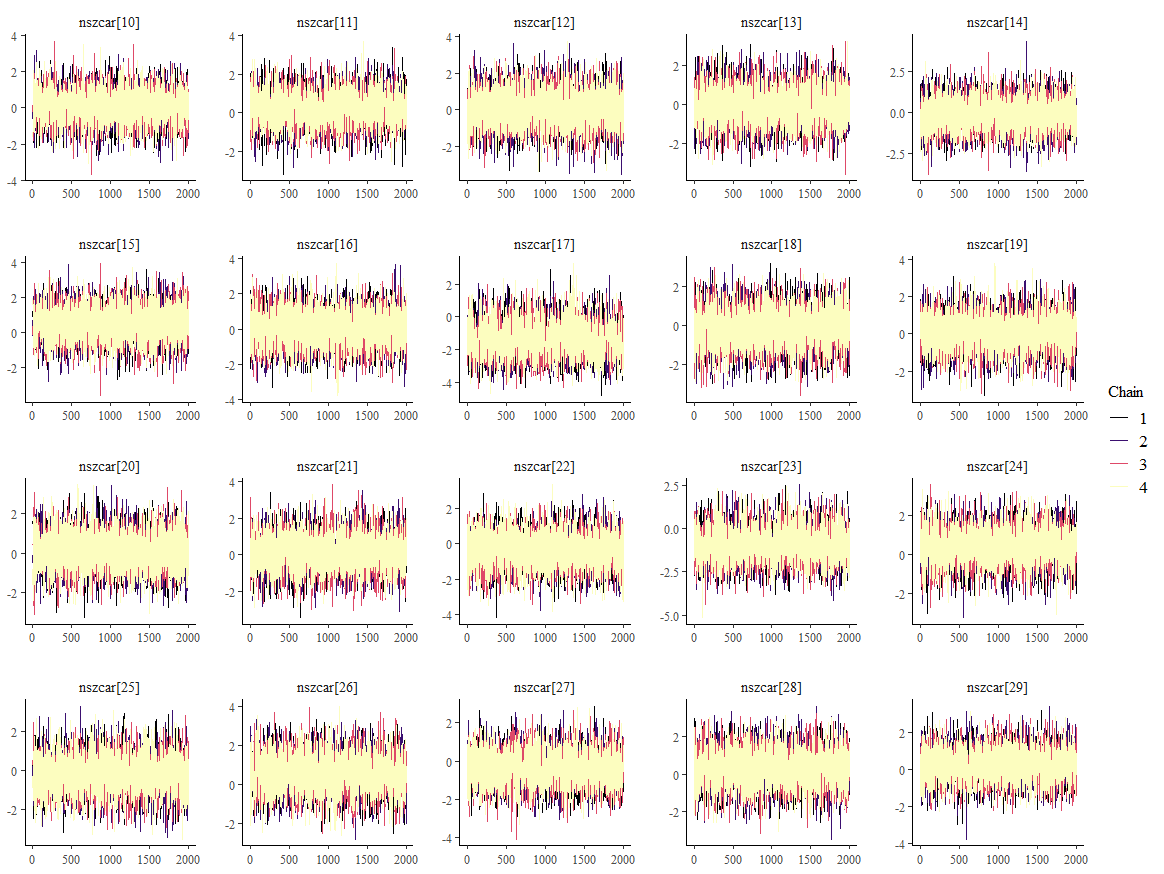


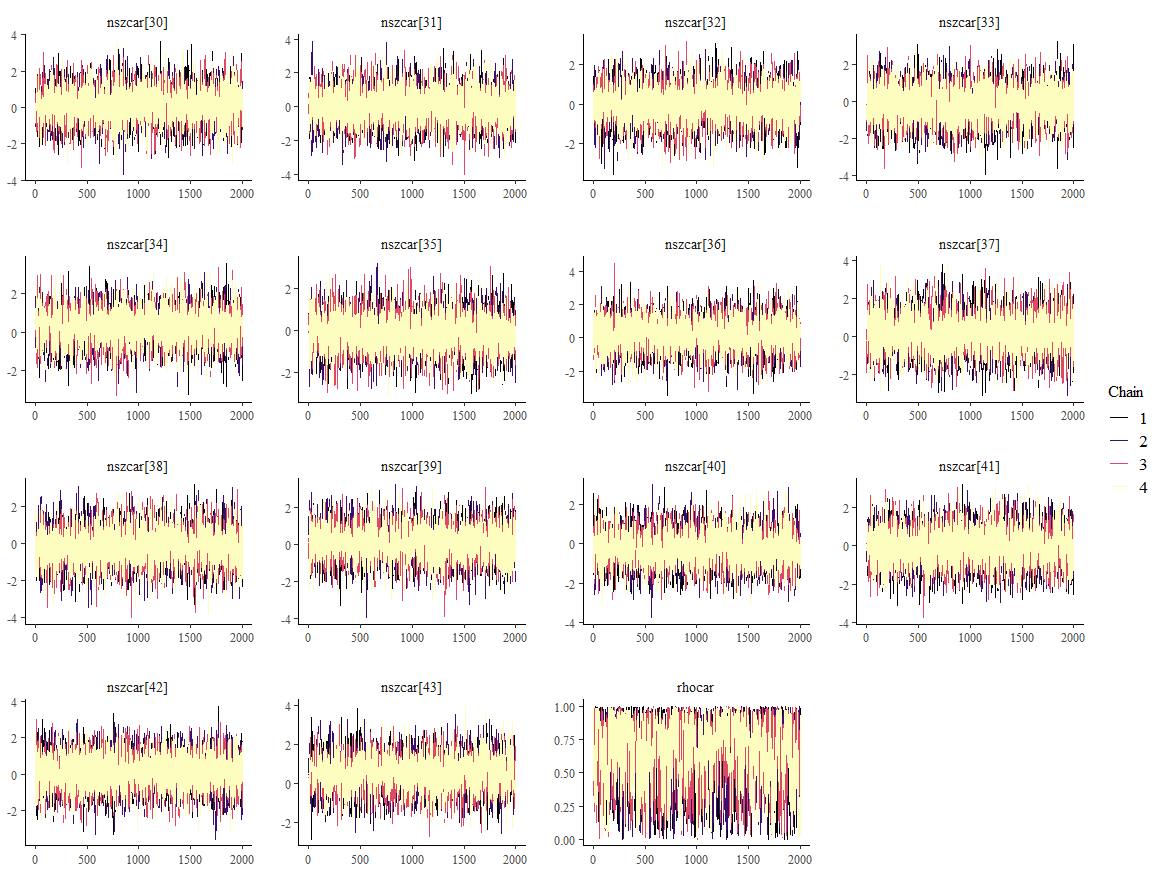


**Figure 4.** Residual plot of the final Bayesian spatial model.


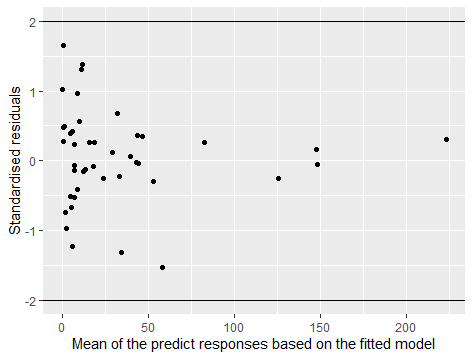


**Figure 5.** Overlaid kernel density of the predictive number of cases (y_rep_) simulated by using the results of the final Bayesian spatial model and the distribution of observed values (y).


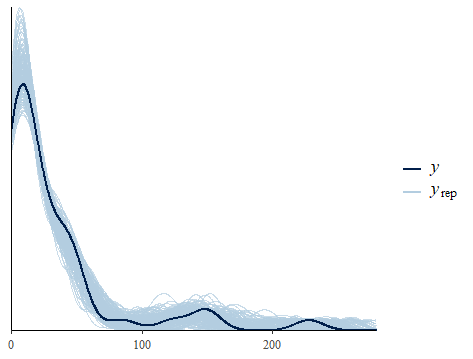


**Figure 6.** Intervals for the predictive number of cases (y_rep_) simulated by using the results of the final Bayesian spatial model (dot: median; inner interval: 50%; outer interval: 90%) and the observed y values.


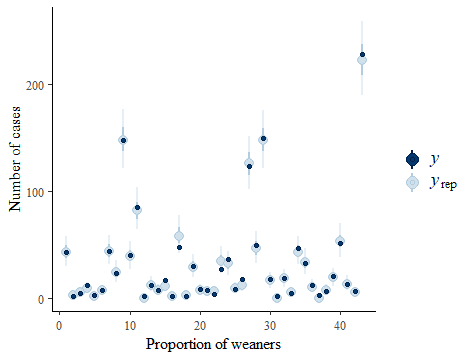

Supplement: Supplementary file 4 [file Data_Sheet_4.docx]
