# Supplementary material for: Spatial Trends in Salmonella Infection in Pigs in Spain
Source: Front Vet Sci. 2020 Jun 23;7:345. doi: 10.3389/fvets.2020.00345 (PMC7325609; doi:10.3389/fvets.2020.00345)

**Supplementary File 5.** Distribution of the density of weaners (per km^2^) and the average of posterior predictive distribution of a Poisson model with the density of weaners as the covariate.

**Figure 1.** The distribution of the density of weaners at the province level in Spain from 2005 to 2019.


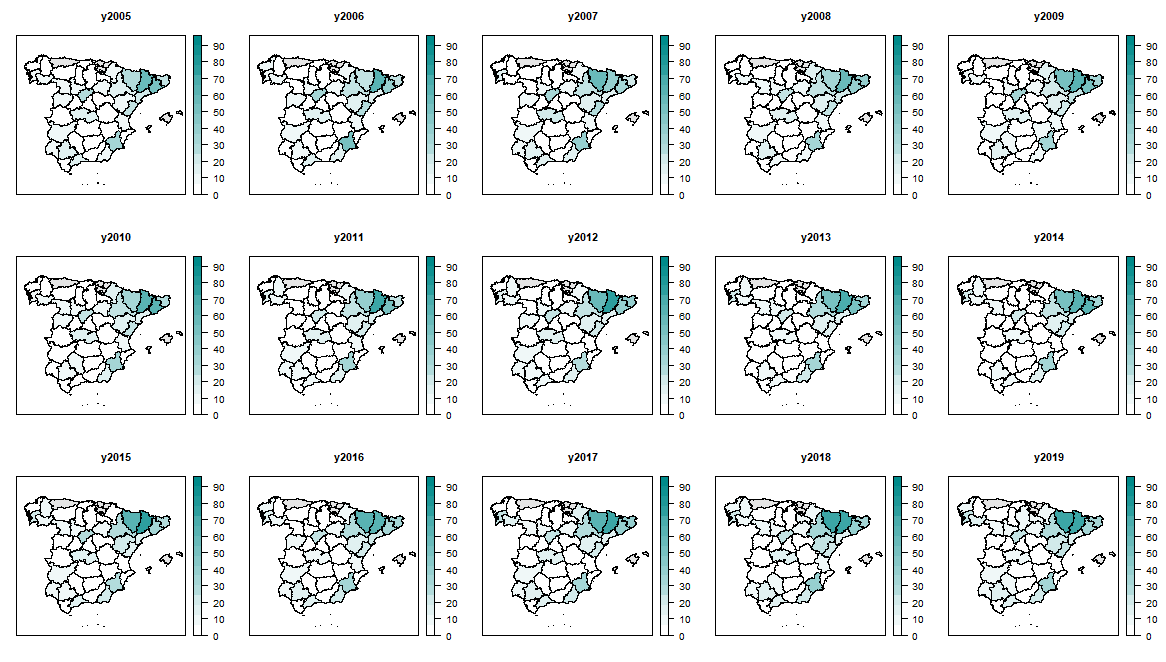


**Figure 2.** The average of posterior predictive distribution of a Poisson model with the density of weaners as the covariate to predict the number *Salmonella*-positive farms at the province level in Spain.


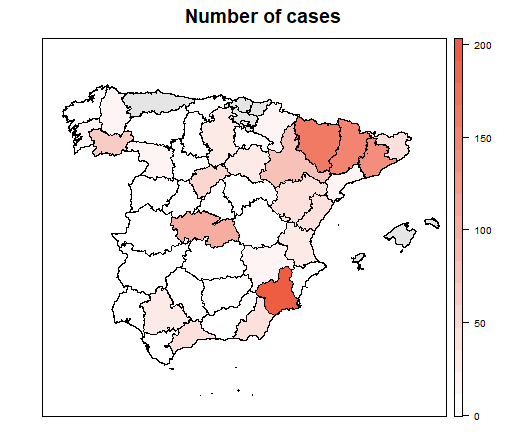

Supplement: Supplementary file 5 [file Data_Sheet_5.docx]
