# Supplementary material for: Spatial Trends in Salmonella Infection in Pigs in Spain
Source: Front Vet Sci. 2020 Jun 23;7:345. doi: 10.3389/fvets.2020.00345 (PMC7325609; doi:10.3389/fvets.2020.00345)
Supplement: Supplementary file 6 [file Table_1.docx]

**Supplementary Table 1.** The numbers of *Salmonella* isolates of different serotypes from samples collected through the Spanish Veterinary Antimicrobial Resistance Surveillance Network programme in Spain from 2003 to 2013, 2015, 2017, and 2019.

| **Serotype** | **Count (proportion)** | **Serotype** | **Count (proportion)** |
| --- | --- | --- | --- |
| Rissen | 313 (22.2%) | Goettingen | 2 (0.1%) |
| Monophasic variant Typhimurium | 265 (18.8%) | Kentucky | 2 (0.1%) |
| Typhimurium | 251 (17.8%) | Mikawasima | 2 (0.1%) |
| Derby | 207 (14.7%) | Mishmarhaemek | 2 (0.1%) |
| Bredeney | 48 (3.4%) | Schwarzengrund | 2 (0.1%) |
| Anatum | 40 (2.8%) | Stanley | 2 (0.1%) |
| Brandenburg | 36 (2.6%) | Toulon | 2 (0.1%) |
| Wien | 35 (2.5%) | Uganda | 2 (0.1%) |
| NA (missing value) | 33 (2.3%) | 16:e,v:- | 1 (0.1%) |
| Kapemba | 19 (1.3%) | 48:z4,z23:-^2^ | 1 (0.1%) |
| 9^1^ | 16 (1.1%) | 9,12:-:1,7 | 1 (0.1%) |
| Brikama | 11 (0.8%) | Agama | 1 (0.1%) |
| London | 9 (0.6%) | Albany | 1 (0.1%) |
| Goldcoast | 8 (0.6%) | Bardo | 1 (0.1%) |
| Infantis | 8 (0.6%) | Duisburg | 1 (0.1%) |
| Montevideo | 7 (0.5%) | Enestudio | 1 (0.1%) |
| Ohio | 7 (0.5%) | Hadar | 1 (0.1%) |
| Enteritidis | 5 (0.4%) | Hessarek | 1 (0.1%) |
| Essen | 5 (0.4%) | Hindmarsh | 1 (0.1%) |
| Muenchen | 5 (0.4%) | Liverpool | 1 (0.1%) |
| Reading | 5 (0.4%) | Livingstone | 1 (0.1%) |
| Bovismorbificans | 4 (0.3%) | Meleagridis | 1 (0.1%) |
| Kedougou | 4 (0.3%) | Newport | 1 (0.1%) |
| Panama | 4 (0.3%) | Paratyphi C | 1 (0.1%) |
| 4,12:d:- | 3 (0.2%) | Regent | 1 (0.1%) |
| Altona | 3 (0.2%) | San Diego | 1 (0.1%) |
| Branderup | 3 (0.2%) | Seremban | 1 (0.1%) |
| Kissi | 3 (0.2%) | Taksony | 1 (0.1%) |
| Senftenberg | 3 (0.2%) | Tennesse | 1 (0.1%) |
| Berta | 2 (0.1%) | Umbilo | 1 (0.1%) |
| Cerro | 2 (0.1%) | Vaganda | 1 (0.1%) |
| Choleraesuis | 2 (0.1%) | Virchow | 1 (0.1%) |
| Give | 2 (0.1%) | Virginia | 1 (0.1%) |

^1^: serotyping not completed

^2^: *Salmonella enterica* subsp. *Arizonae* serovar 48:z4,z23:-
